# Chromosome Compaction by Active Loop Extrusion

**DOI:** 10.1016/j.bpj.2016.02.041

**Published:** 2016-05-24

**Authors:** Anton Goloborodko, John F. Marko, Leonid A. Mirny

**Affiliations:** 1 Department of Physics, Massachusetts Institute of Technology, Cambridge, Massachusetts; 2 Department of Molecular Biosciences and Department of Physics and Astronomy, Northwestern University, Evanston, Illinois; 3 Institute for Medical Engineering & Science, Massachusetts Institute of Technology, Cambridge, Massachusetts

## Abstract

During cell division, chromosomes are compacted in length by more than a 100-fold. A wide range of experiments demonstrated that in their compacted state, mammalian chromosomes form arrays of closely stacked consecutive ∼100 kb loops. The mechanism underlying the active process of chromosome compaction into a stack of loops is unknown. Here we test the hypothesis that chromosomes are compacted by enzymatic machines that actively extrude chromatin loops. When such loop-extruding factors (LEF) bind to chromosomes, they progressively bridge sites that are further away along the chromosome, thus extruding a loop. We demonstrate that collective action of LEFs leads to formation of a dynamic array of consecutive loops. Simulations and an analytically solved model identify two distinct steady states: a sparse state, where loops are highly dynamic but provide little compaction; and a dense state, where there are more stable loops and dramatic chromosome compaction. We find that human chromosomes operate at the border of the dense steady state. Our analysis also shows how the macroscopic characteristics of the loop array are determined by the microscopic properties of LEFs and their abundance. When the number of LEFs are used that match experimentally based estimates, the model can quantitatively reproduce the average loop length, the degree of compaction, and the general loop-array morphology of compact human chromosomes. Our study demonstrates that efficient chromosome compaction can be achieved solely by an active loop-extrusion process.

## Introduction

During cell division, interphase human chromosomes are compacted in length by more than a 100-fold into the cylindrical, parallel-chromatid metaphase state. Several lines of evidence suggest that this compaction is achieved via formation of loops along chromosomes (1, 2). First, chromatin loops have long been observed via electron microscopy (2, 3, 4). These observations served as a basis for the radial-loop models of the mitotic chromosome (4) and are consistent with optical imaging data (5). Second, theoretical studies showed that compaction into an array of closely stacked loops could explain the observed shape, the mechanical properties, and the degree of compaction of mitotic chromosomes (6, 7, 8). More recently, the general picture of mitotic chromosomes as a series of closely packed chromatin loops was supported by Hi-C experiments, which measured the frequency of physical contacts within chromosomes (9). The same study independently confirmed the ∼100 kb length of the chromatin loops.

The mechanism underlying compaction of chromosomes into a stack of loops is unknown. Several lines of evidence suggest that this compaction cannot be achieved by simple mechanisms of chromatin condensation, e.g., poor solvent conditions, or nonspecific chromatin cross-linker proteins. First, the loops are formed overwhelmingly within individual chromatids. Different chromosomes and sister chromatids are not extensively cross linked to each other as would tend to happen during nonspecific condensation, but instead become individualized during the compaction process. Second, loops are arranged in essentially genomic order and are nonoverlapping (9), without the strong overlap of loops that would be expected from nonspecific cross linking. Finally, metaphase chromosomes compact into elongated structures with a linear arrangement of loops along the main axis. A cross-linking agent would generate surface tension and shrink chromosomes into spherical globules with a random spatial arrangement within a globule (6, 10, 11). In fact, the term “condensation”, which generally refers to the effects of chemical interactions driving phase separation and surface tension, is inappropriate for description of mitotic chromosome compaction where neither effect occurs. Chromatin is clearly being actively compacted during mitosis.

An alternative hypothesis is that chromosomes are compacted by enzymatic machines that actively extrude chromatin loops (12, 13). When these enzymes bind to chromosomes, they first link two adjacent sites, but then move both contact points along the chromosome in opposite directions, so that they progressively bridge more distant sites (12). Loop-extruding functions have been observed for other enzymes acting on naked DNA (14, 15, 16, 17). Condensin complexes, which play a central role in chromosome compaction (18) and are present in all domains of life (19), are likely to be a key component of such loop-extruding factors (LEFs). A key question is whether LEFs alone are sufficient to drive formation of arrays of nonoverlapping loops essential for linear compaction of chromatids, or if other factors are required, e.g., to define the loop bases.

The previous study of Alipour and Marko (13) introduced a quantitative model of loop extrusion and considered dynamics of solvent-exchanging LEFs on a short chromosomal segment. They found that formed chromatin loops can be stabilized by multiple stacked LEFs, making loops robust to exchange of individual LEFs. A small system size, however, prevented them from obtaining a complete picture of self-organization. The remaining key question is whether LEFs alone are sufficient to form arrays of nonoverlapping loops on a long chromosome or if other factors are required, e.g., to define the loop bases.

In this article, we model the collective action of LEFs that dynamically exchange between the nucleoplasm and chromatin fiber. We find that LEFs self-organize into a dynamic array of consecutive loops, which has two distinct steady states: a sparse state where loops are separated by gaps and provide moderate compaction; and a dense state where jammed LEFs drastically compact a long chromatin fiber. These states can be described by a simple analytical model of loop dynamics. We show how the macroscopic characteristics of the loop array are determined by the microscopic properties of the LEFs and their abundance, and we demonstrate that efficient chromosome compaction can be achieved solely by LEFs.

## Materials and Methods

Simulations were performed using the Gillespie algorithm (13, 20). The Python code performing the simulations of loop extrusion and the data analysis is available online at http://github.com/golobor/loop-extrusion-1d. See the Supporting Material for details of simulations.

## Results

### Model for LEFs on a long chromatin segment

**Figure 1:**
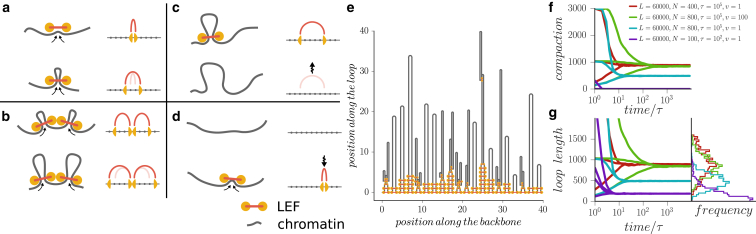
Simulations of chromosome compaction by loop extruding factors (LEFs). The action of LEFs can be simulated using a one-dimensional lattice model with four dynamic rules (*a*–*d*): (*a*) LEFs extrude loops by moving the two connected heads along the chromosome, (*b*) LEF heads block each other, (*c*) LEFs dissociate from chromatin, and (*d*) LEFs in the solution rebind to the chromosome. (*e*) Simulations show that LEFs can fold a chromosome into an array of consecutive loops. The diagram shows the loops formed by LEFs in a simulation with *L* = 2000, *N* = 200, *τ* = 450, and *v* = 1 after 45,000 time steps. (*f* and *g*) The system of LEFs on a long chromatin fiber converges to a steady state. The steady distribution of loop sizes and the degree of compaction depends on the control parameters, but is independent of initial state. Results are shown for different simulation parameters and starting conditions; data for each curve is averaged over 10 simulation replicas. To see this figure in color, go online.

To understand the dynamics of loop formation and chromatin compaction by LEFs, we carried out stochastic simulations of the process shown in Fig. 1 (13). We focus on the organization and dynamics of loop formation and dissolution without considering three-dimensional organization of the chromatin fiber and assume that emerging topological conflicts can be resolved by topoisomerase II enzymes active during metaphase compaction.

We consider a single piece of chromatin fiber of length *L*, occupied by *N* LEFs. We model a LEF very generally as having two heads connected by a linker. The LEF heads bind to nearby sites along the chromatin fiber and proceed to slide away from each other stochastically with an average velocity *v*, thus extruding a loop with rate 2*v* (Fig. 1 *a*). When the heads of two neighboring LEFs collide, they block each other and halt (Fig. 1 *b*), while the other heads of each LEF remain unperturbed and continue loop extrusion. Below we show that this assumption of uncoupled dynamics of the two heads is critical, as LEFs with coupled dynamics of heads fail to generate a gapless array of loops.

For the LEFs to be able to organize robust loop domains, it is essential that they be able to relocate via unbinding and rebinding (13). We allow each LEF to dissociate at a rate 1/*τ*, which is independent of their state and location (Fig. 1 *c*). However, we maintain a constant number of LEFs on the chromosome; upon dissociation of one LEF, another LEF binds a random site elsewhere on the chromosome, where it begins to extrude a new loop (Fig. 1 *d*). When the assumption of a constant number of bound LEFs is replaced with explicit modeling of dissociation and association processes, the main results of simulations remain unaffected (Fig. S13).

The model is fully determined by the four parameters (*L*, *N*, *v*, *τ*), of which *N* and *τ* can be estimated from experimental studies of condensins (21, 22, 23). We divide the chromosome into *L* = 6 × 10^4^ sites, so that each site can be occupied by one LEF head. With each site roughly corresponding to a nucleosome with a DNA linker (∼200 bp or ∼10 nm, a fraction of a size of a condensin complex), our simulated chromosome corresponds to ∼12 Mb of chromatin fiber.

### LEFs can generate a tightly stacked loop array and strong chromosome compaction

In initial simulations we observed that the LEFs generated tightly stacked loops with a high degree of chromatin compaction, despite their constant dissociation (Fig. 1, *e*–*g*; Movie S1). To test that this was a steady state rather than a frozen (glassy) configuration, we performed simulations 10 times longer than the apparent time needed to reach the steady state, and used a broad range of initial conditions (Supporting Material). Simulations converged to states with degree of compaction and distribution of loop size that depended on the control parameters, but were independent of initial states (Fig. 1, *f* and *g*, and the Supporting Material), providing further support to the existence of a well-defined, loop-stacked steady state.

### Two characteristic lengths control whether LEFs form dense or sparse chromatin loops

**Figure 2:**
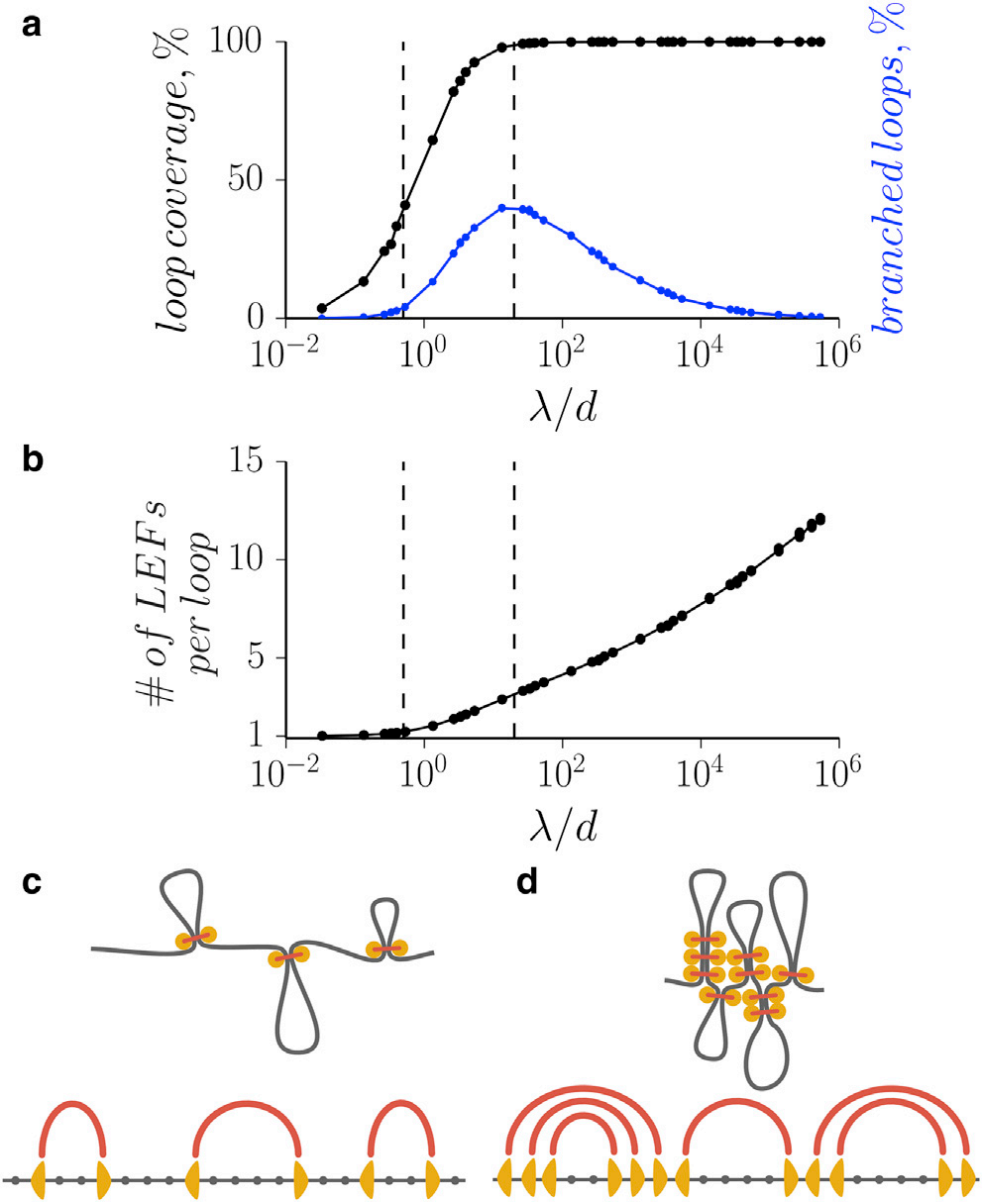
Simulations of LEFs reveal two distinct steady states. (*a* and *b*) The properties of loop arrays formed by LEFs, such as the portion of the chromosome extruded into loops, the portion of branched loops, and the number of LEFs per loop, depend on the dimensionless ratio *λ*/*d*. This ratio defines the two steady states of the system: (*c*) the sparse state (*λ*/*d* ≪ 1), where the loops are supported by single LEFs and separated by big loop-free gaps; and (*d*) the dense state (*λ*/*d* ≫ 1), where the whole chromosome is extruded into an array of consecutive loops supported by multiple LEFs. In both steady states, the loops are not branched (*a*). The vertical dotted lines at *λ*/*d* = 0.5 and 20 roughly show the transition region. To see this figure in color, go online.

To understand how the microscopic characteristics of the LEFs control the compaction process, we performed simulations systematically exploring the control parameter space. This revealed that there are two distinct steady states of loop-extrusion dynamics in the model (Fig. 2): 1) a sparse, poorly compacted state, where loops are formed by single LEFs and separated by gaps (Fig. 2 *c*); and 2) a dense state, where the chromosome is compacted into an array of consecutive loops, each having multiple LEFs at its base (Fig. 2 *d*).

In the sparse state, LEFs do not efficiently compact chromosomes, because even a small fraction of fiber length remaining unextruded in the gaps between loops prevents efficient linear compaction (Fig. S7). In the dense state, however, the whole chromosome is folded into a gapless array of loops, with the end of one loop adjacent to the beginning of the following one (Fig. 2). Such organization was found to be essential to achieve agreement with Hi-C data for mitotic chromosomes (9). Below, we show that realistic LEF abundance (one condensin per 10–30 kb) can give rise to loop sizes of ∼80–120 kb consistent with mitotic Hi-C and earlier direct measurements (3, 22, 23, 24, 25) and inferred from Hi-C data (9). These findings suggest a dense state as an attractive model of chromosome compaction.

Two steady states arise from the interplay of two length scales characterizing the LEFs: (1) processivity *λ* = 2*vτ*, the average size of a loop extruded by an isolated LEF during its residency time on chromatin; and (2) the average linear separation between LEFs *d* = *L*/*N* (Fig. 2 *a*). When *λ*/*d* ≪ 1, the system resides in the sparse state: LEFs work in isolation, a small fraction of the chromosome is extruded into loops, and large gaps between them prevent efficient compaction. In the opposite dense case, when *λ*/*d* ≫ 1, the whole chromosomal fiber is extruded into loops, leading to a high degree of compaction. When the loop coverage is plotted as a function of *λ*/*d* rather than individual parameters, the curves collapse into a single transition curve, which is indicative of the central role of *λ*/*d* in controlling the compaction (Fig. S1).

### Loop organization and dynamics are distinct in the sparse and dense steady states

To understand the process of chromosome compaction by LEFs, we consider the dynamics of loop formation and disassembly. In the sparse state, LEFs rarely interact; each loop is extruded by a single LEF and it disappears once the LEF dissociates, leading to a highly dynamic state with a rapid (∼*τ*) turnover of loops (Fig. S2 *a*). Because LEFs extrude loops continuously and the distribution of their residence times is exponential, the distribution of loop size is exponential too (Fig. S3 *a*).

**Figure 3:**
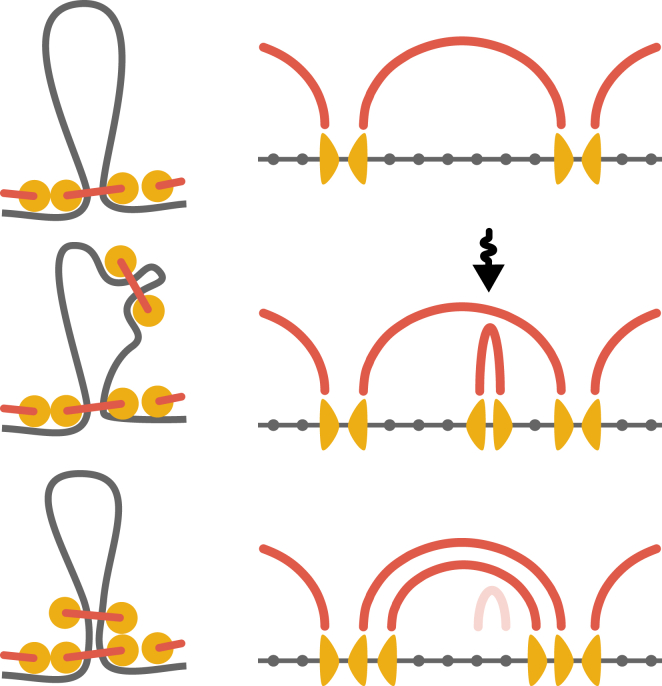
The mechanism of loop reinforcement in the dense state. Upon binding to an existing loop, a LEF reextrudes it and stacks on top of the LEFs already supporting the loop. To see this figure in color, go online.

Two aspects of the loop organization control the dense state dynamics: (1) loops have no gaps between each other; and (2) individual loops are reinforced by multiple LEFs, i.e., several LEFs are stacked on top of each other at the base of a loop (13). Both phenomena result from the competition for chromosomal fiber among abundant LEFs. The gaps disappear because, in the dense state, LEFs have enough time to extrude all available fiber until colliding with adjacent LEFs. For gaps to disappear, two LEF heads should have uncoupled dynamics, i.e., when one is blocked, the other continues extruding. Simulations with coupled kinetics of heads cannot produce a gapless array of loops (Fig. S14). Abundant collisions lead to a nonexponential distribution of loop sizes (Fig. S3 *a*). Loop reinforcement is also caused by LEF collisions (Fig. 3): every time a new LEF binds within an existing loop, it reextrudes this loop until colliding with the LEF residing at the loop base. As a result, each loop is stabilized by multiple LEFs at its base. The absence of gaps and the reinforcement of loops preserve the structure of loops on timescales *t* ≫ *τ* (Fig. S2 *b*): loops cannot grow because their LEFs are blocked by the neighbors, and they do not disband when individual LEFs dissociate, as remaining LEFs support them. Thus, the loops of a LEF-*n* compacted chromosome are maintained despite continuously exchanging LEFs.

### Steady-state loop dynamics is controlled by competition between loop death and division

To develop an analytical model of the system’s steady state, we consider its dynamics. Loops in the dense state are not completely static: two stochastic processes, i.e., loop death and loop division, change the structure of the loop array and drive self-organization of the steady state.

**Figure 4:**
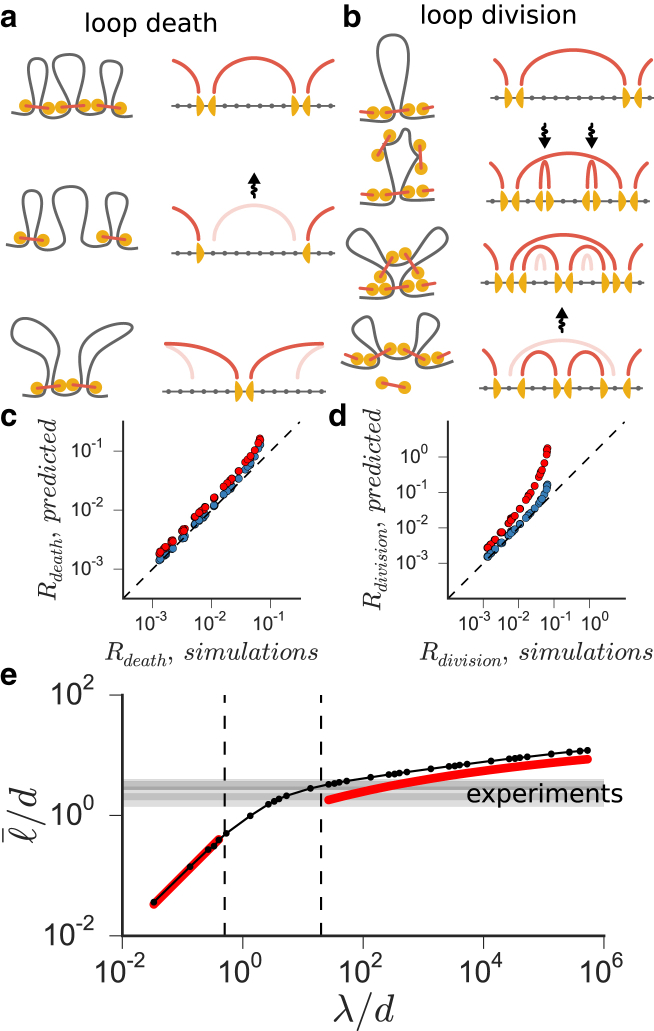
The model of loop death and division explains the origin of the dense steady state. (*a*) Loops occasionally disassemble when the number of reinforcing LEFs fluctuates to zero. The chromatin of the disassembled loop is immediately extruded into the adjacent loops. (*b*) A loop splits upon simultaneous landing of two reinforcing LEFs. The rates of loop death (*c*) and division (*d*) in the dense state can be estimated using simple analytical formulas (*red dots*) or more accurate computational models (*blue dots*). (*e*) In the dense state, the steady-state balance between loop death and division provides an approximate analytical expression for the average loop length (the *red line*). In the sparse state, the average loop length is predicted to be equal *λ* (the *red line*). Both predictions agree well with the simulations (the *black line*). The four horizontal overlapping gray bands show the available independent experimental estimates of $\bar{\ell }/d$ in mitotic human chromosomes: $\bar{\ell }$ = 42–70 kb (3), 54–112 kb (25), 80–90 kb (24), 80–120 kb (9), and *d* ≈ 30 kb (22). To see this figure in color, go online.

A loop dies when the number of LEFs at its base supporting it fluctuates to zero. When all LEFs dissociate, neighboring LEFs become unblocked and extrude the released fiber into their own loops (Fig. 4 *a*). We compute the rate at which a stack of LEFs supporting a loop of size can stochastically fluctuate to zero. The stack can shrink due to LEFs dissociation (at rate *n*/*τ*) and can grow due to association of new LEFs to the loop (at rate ∼ℓ(N/L)τ=(1/τ)(ℓ/d)). Fluctuations of the LEF stack size are equivalent to the stochastic immigration-death process, for which the rate of fluctuation to zero can be computed as $R_{\text{death}}∼\left(1/\tau \right)\left(\ell /d\right)e^{-\ell /d}$ (see the Supporting Material) (26).

A loop divides into two smaller loops when two LEFs land within a single loop almost simultaneously and extrude two smaller consecutive loops (Fig. 4 *b*). These newly created loops become subsequently reinforced by other LEFs that land onto them. The original parent loop, on the contrary, is effectively cut off from the supply of reinforcing LEFs, and disintegrates on a timescale ∼*τ*, with the two child loops taking its place. The rate-limiting process for loop division is the landing of two LEFs onto the same loop, giving an estimate for the rate of division: $R_{\text{division}}∼\left(1/\tau \right){\left(\ell /d\right)}^{3}\left(d/\lambda \right)$ (see the Supporting Material). These scaling laws accurately predict the dynamics of loop birth and death (Fig. 4, *c* and *d*)

In the steady state, the number of loops is approximately constant. By equating the rate of loop creation by division to the rate of loop death, the average loop size is obtained as:

(1) $$\bar{\ell }\approx 2d\;W\left(\sqrt{\frac{3\lambda }{8d}}\right)∼d\;\text{ln}\left(\frac{\lambda }{d}\right),$$

and the average number of LEFs per loop is:

(2) $$\bar{n}\approx 2\;W\left(\sqrt{\frac{3\lambda }{8d}}\right)∼\text{ln}\left(\frac{\lambda }{d}\right),$$

where *W*(*x*) is the Lambert *W* function.

Our analytical model agrees with simulations (Fig. 4 *e*) and explains how the number of LEFs and their microscopic properties affect the morphology of compacted chromosomes. First, Eqs. 1 and 2 show that *λ*/*d* is the key control parameter of the system, which determines not only the state of the system (sparse versus dense), but also loop sizes and the degree of loop reinforcement in the dense state. Using these scaling laws plus available experimental data, we can estimate LEF processivity and dynamic state for human metaphase chromosomes. The average loop length has been estimated by microscopy and via modeling of Hi-C data as ℓ=80−120kb (3, 9, 24, 25). The spacing between bound condensin molecules was measured as *d* = 30 kb (22). Using these values, we obtain a range of ℓ/d≈3, shown in Fig. 4 *e*, which corresponds to *λ*/*d* ≈ 20. These values shows that human mitotic chromosomes operate at the lower bound of the dense state, have each loop reinforced by *n* ≈ 3 LEFs, and human LEFs have a processivity *λ* ≈ 600 kb.

Second, our analysis allows us to compute the degree of chromosomal compaction by LEFs. Because the length of a compacted chromosome in the gapless dense state equals the sum of the widths of the loop bases, *a* (Fig. 1 *e*), the coefficient of chromosomal compaction is $c=\bar{\ell }/a$. While addition of extra LEFs leads to better loop reinforcement, it also makes loops shorter (ℓ∼lnN/N) and thus reduces the degree of chromosomal compaction (Fig. S7). For the loop base size close to the chromatin fiber diameter *a* = 10–20 nm, we obtain the degree of compaction $\bar{\ell }/a=4-12\;\text{kb/nm}$. Interestingly, our estimate for the compaction achieved through folding of a chromosome into a gapless array of loops is in good agreement with the experimentally measured degree of human chromosome compaction in midprophase (∼6 kb/nm) (27).

Third, our model predicts how the loop array morphology changes in response to biological perturbations. Specifically, factors that decrease the speed of loop extrusion *v* or reduce LEF residence time *τ* will decrease the processivity *λ* and thus decrease the average loop size, degree of loop reinforcement, and the degree of chromatid compaction. The effects of LEF overexpression depend on the state of the system: for sparse loop arrays, it does not affect the average loop size and only increases the number of loops and, thus the degree of compaction. In the dense systems, LEF overexpression decreases the average loop size and degree of compaction, but increases the degree of loop reinforcement.

Finally, this analytical model shows how LEFs robustly self-organize chromosomes into a globally stable steady state. The rates of death and division $R_{\text{death}}$ and $R_{\text{division}}$ scale differently with the loop length: large loops are more likely to divide into smaller ones $\left(R_{\text{death}}∼\ell^{3}\right)$, and smaller loops are more likely to die $\left(R_{\text{death}}∼\ell e^{-\ell }\right)$, allowing neighboring loops to grow. This negative feedback drives the system to a steady state with a relatively narrow distribution of loop lengths. These results indicate that loop sizes and hence chromosome diameter and length will be sensitive to concentrations of LEFs while the overall morphology as a gapless array of consecutive loops will remain unchanged as long as the system remains in the dense state.

## Discussion

The model of loop-extrusion (12, 13) provides a resolution of the puzzle of how roughly 50-nm-sized enzyme complexes can drive the regular organization of a chromosome at scales well beyond a micron, as occurs in eukaryote cells during mitosis. A fundamental problem with almost any mechanism based on nonspecific crosslinking of chromatin fibers is that chromosomes will end up cross linked together; the mechanism of loop extrusion avoids this fate by having LEFs bind to chromatin at one location and then actively extrude loops without the possibility of forming interchromosome attachments.

Through unbinding, rebinding, and reextrusion, enzymatic machines of this type gradually build larger loops supported by multiple LEFs, eventually reaching a steady state. We find that LEFs alone are sufficient to form arrays of nonoverlapping loops on a long chromosome, without a need for defined positions of loop bases, boundary elements (13), or additional sequence information. Additional activity of topoisomerase II, however, may be required to resolve possible topological entanglements during chromosome compaction. A key feature of our model is that the compaction process proceeds by a combination of stochastic loop death and division events, which gradually but not strictly monotonically lead to a highly compacted chromosome.

The lengthwise compaction driven by LEFs is distinct from the usual polymer condensation occurring under poor solvent conditions. Unlike the proposed linear compaction generated by LEFs, nonspecific adhesion of chromatin fibers to one another would generate surface tension, driving adhesion of chromosomes together into spherical masses of chromatin (11), increasing entanglement and working against chromosome segregation and individualization (8, 13). An important feature of the LEF-compacted state is that despite its robust structure, it is entirely dependent on DNA connectivity; intermittent cleavage of DNA alone can lead to dissolution of the entire chromosome, as has been observed experimentally (28, 29).

We emphasize that in the compacted steady state the loops have a well-defined size, and that inside the chromosome the LEFs establish internal tension, rather than the surface tension generated by nonspecific crosslinking. This internal tension is an essential contributor to the uniform folding and well-regulated cylindrical morphology of chromatids, and generates repulsive forces between folded chromosomes essential to segregation of sister chromatids and individualization of different chromosomes (6).

Note that achieving a compacted steady state by this mechanism can be a slow process, i.e., it would require up to ≤10*τ*, while the turnover rate *τ* for condensin was measured to be at least a few minutes (21). However, we found that gradual or stepwise loading/activation of LEFs can lead to a significant speedup of the process (see the Supporting Material). For the optimal activation rate, the compact steady state can be achieved in a fraction of time *τ* (see Fig. S9). A sign of this dynamics occurring in vivo is the gradual activation of condensin by phosphorylation during early prophase (30, 31).

On the other hand, if there are too few LEFs, we have found that a distinct, disordered, poorly compacted chromosome steady state occurs. This outcome has been observed in experiments where condensins were interfered with, both in cells (32) and in *Xenopus* egg extracts (18, 32). Modulation of chromosome structure also has been observed to occur through development, for example in *Xenopus*, where mitotic chromosomes become gradually shorter and fatter with maturation (33); this gradual change in chromosome morphology could be due to changes in LEF amount or activity with development.

While the mechanisms of loop extrusion remain unknown, a relative simple molecular organization of a protein complex could produce loop-extruding activity. A LEF composed of two connected heads, each able to move along chromatin fiber processively, can achieve a loop extrusion activity. In fact, relative dynamics of the two heads (motors) does not have to be coordinated: Of four possible relative orientation of heads’ directions (→→, ←←, →←, ←→), two (→→, ←←) produce LEFs that slide along chromatin without loop extrusion, one (→←) makes LEFs with heads pushing against each other and thus stuck on chromatin, and the last one (←→) makes LEFs with heads moving away from each other and thus extruding loops.

Moreover, uncoupled dynamics of LEF heads is essential to produce a gapless array of loops: when one head is blocked, the other needs to proceed with loop extrusion. Our simulations show that if both heads stop upon blocking of one, the gapless array cannot be formed (Fig. S14). It remains to be seen how these functions are implemented in structural maintenance of chromosome complexes (cohesins and condensins) that have P-loop-containing ATPase domains homologous to SF1 and SF2 helicases and translocases (e.g., FtsK), and which can translocate along DNA without unwinding the duplex (34).

We note that our model does not discern between condensin I and condensin II, and that the considered compaction process is the prophase compaction driven by condensin II (32). Experiments aimed at disrupting LEFs would perhaps be best targeted at condensin II; however, other proteins may be involved, as condensin II by itself is not thought to have motor function. Intriguingly, the motor KIF4A has been shown to be involved with mitotic chromosome compaction (35); it is conceivable that condensins are somehow aided in a LEF function by a separate motor molecule such as KIF4A. Alternately, condensins may be able to cooperatively organize to generate contractile LEF behavior—for example, by directional polymerization (13).

## Author Contributions

A.G. developed the computational model, analyzed data, developed the analytical model, and wrote the article; J.F.M. developed the concept of loop extrusion in mitotic chromosome compaction, and wrote the article; and. L.A.M. supervised modeling, interpreted the data, developed the analytical model, and wrote the article.

## Supporting Citations

References (36, 37) appear in the Supporting Material.

## Supporting Material

Document S1. Supporting Materials and Methods, Supporting Results, and Figs. S1–S15

Movie S1. Representative Simulation of Chromosome Compaction by Loop Extruding Factors

Document S2. Article plus Supporting Material
