# Supplementary material for: Chromosome Compaction by Active Loop Extrusion
Source: Biophys J. 2016 May 24;110(10):2162–8. doi: 10.1016/j.bpj.2016.02.041 (PMC4880799; doi:10.1016/j.bpj.2016.02.041)
Supplement: Document S1. Supporting Materials and Methods, Supporting Results, and Figs. S1–S15 [file mmc1.pdf]

Biophysical Journal, Volume *110*

Supplemental Information

Chromosome Compaction by Active Loop Extrusion

Anton Goloborodko, John F. Marko, and Leonid A. Mirny

Biophysical Journal

Supporting Material

Chromosome Compaction by Active Loop Extrusion

Anton Goloborodko,[1] John F. Marko,[2] and Leonid A. Mirny[1,3,]*

[1]Department of Physics, Massachusetts Institute of Technology, Cambridge, Massachusetts; [2]Department of Molecular Biosciences and Department of Physics and Astronomy, Northwestern University, Evanston, Illinois; and [3]Institute for Medical Engineering & Science, Massachusetts Institute of Technology, Cambridge, Massachusetts

*Correspondence: leonid@mit.edu

Supporting material

Mitotic chromosome compaction via active loop extrusion

Contents

1 Materials and methods. 3

2 The two regimes of LEFs on a chromosome. 3

2.1 Loops have different dynamics in the sparse and dense regimes. . 4

2.2 The distribution of loop lengths is exponential in the sparse regime and normal in the dense regime. 5

2.3 Gaps disappear exponentially with λ/d. 6

3 The theory of self-organization of loop arrays in the dense regime. 7

3.1 In the dense regime, loops have stable lengths and fluctuating numbers of reinforcing LEFs. 7

3.2 The number of LEFs in a loop is approximately Poisson-distributed around ℓ/d. 7

3.3 The lifespan of reinforced loops increases exponentially with their length. 9

3.4 Loops in the dense regime stochastically divide in two. 10

3.5 The balance between loop death and division gives rise to the steady state of the dense regime. 10

3.6 Maximal lengthwise compaction is achieved on the lower border of the dense regime. 12

3.7 The maximal degree of total compaction depends on the length of the chromosome and the size of LEFs. 13

3.8 Gradual activation of LEFs speeds up convergence to the steady state. 14

4 Corrections to the theory of self-organization of loop arrays. 18

4.1 The theory of stochastic processes provides an exact expression for the lifespan of loops. 18

4.2 Loops supported by a single LEF can die immediately. 20
4.3 Accounting for immediate death gives a simple and accurate estimate for the rate of loop death. 21
4.4 The lifespan of a loop after interruption of reinforcement is approximately Gumbel-distributed. 22
4.5 Selection for the minimal daughter loop size slows down loop division. 23
4.6 Immediate death of daughter loops slows down loop division. . . 24
4.7 Loop size selection and immediate death affects daughter loops independently. 25
4.8 Fluctuations of the number of LEFs bound to the chromosome does not affect the properties of the steady state. 26
4.9 Closing gaps between the loops requires independent extrusion on the two sides of a LEF. 27

5 Glossary and mathematical notation **29**

References **31**

1 Materials and methods.

We study the action of loop extruding factors (LEFs) using the previously described model [1]. In this model, we model a chromosome as a one-dimensional lattice with L sites with N LEFs. Each LEF is represented as a pair of "heads", each occupying an individual site on the chromosome. The positions of LEF heads are stochastically updated using the Gillespie algorithm with four rules:

1. The two heads of each LEF stochastically step away from each other with the average rate v.
2. The heads of different LEFs cannot step over each other and thus stop extrusion upon reaching another LEF. However, the two heads of the same LEF extrude loops independently and if one head of a LEF is blocked, another head continues extrusion.
3. LEFs stochastically unbind from the fiber with the rate of $\frac{1}{\tau}$, where τ is the average residence time.
4. Free LEFs immediately rebind to the chromatin fiber at a random uniformly chosen pair of adjacent sites.

In this study we modeled 12Mb of chromatin fiber, close to the size of the smallest human chromosomal arm 21p (12.7 Mb). Without loss of generality, we divided the fiber into a lattice of $L = 60000$ cells of 200 bp each, roughly the size of a nucleosome with a DNA linker. We simulated systems with $N =$ 100, 400, 800, 1200 and 1600 LEFs, where 400-1200 LEFs corresponded to the experimental estimates of the abundance of condensin in mitotic human cells (1 per 10-30kb) [2, 3]. The speed of extrusion v varied in a broad range between 1 and 100 sites per time unit and the residence time τ varied between 10 and 10^5 time units. At the beginning of each simulation, LEFs were distributed randomly along the chromosome with both heads in adjacent lattice sites. We simulated each system for $10^4 \cdot \tau$ units of time, with 10 simulations per each parameter set.

We found that the average loop length $\bar{\ell}$ in the steady state did not depend on the initial positions of LEFs, with only 1 out of 75 tested parameter sets failing the Bonferroni-corrected one-way ANOVA comparison of $\bar{\ell}$ between ten randomly initiated replicas. Additionally, we found that the same final values of $\bar{\ell}$ were achieved if the system was initiated with 20 or 60 loops of equal length, each supported by closely stacked LEFs.

2 The two regimes of LEFs on a chromosome.

We found that the system of LEFs has two distinct regimes: the sparse regime, where loops are formed by individual LEFs and are separated by gaps, and the dense regime, where loops are supported by multiple LEFs and cover the chromosome completely. The transition between the two regimes occurs when

we increase the parameter $\frac{\lambda}{d}$, where $\lambda = 2v\tau$ is the LEF *processivity*, i.e. the average length of a loop extruded by an obstructed LEF over its residence time on chromatin and $d = \frac{L}{N}$ is the average linear *separation* between LEFs. Our simulations suggested that this ratio, and not each of the parameters alone, determines the average loop coverage, i.e. the portion of chromatin extruded into loops (Figure S1).

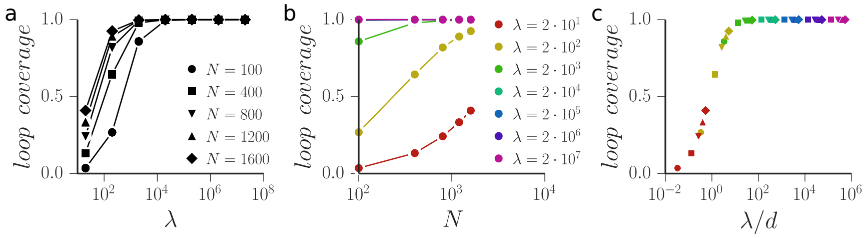

Figure S1: Loop coverage as (a) a function of the LEF processivity λ for different numbers of LEFs N and (b) as a function of N for several values of λ. (c) The curves collapse when plotted relative to the ratio $\lambda/d = \lambda N/L$.

2.1 Loops have different dynamics in the sparse and dense regimes.

We found that loops in the two regimes of LEFs display very different dynamics (Fig. S2). As a proxy for the timescale of loop stability we measured the autocorrelation time of the LEF footprint on chromatin. This measure allowed us to estimate the characteristic times of change in loop structures across multiple orders of magnitude. In the sparse regime, autocorrelation time is much shorter than τ, inversely proportional to the speed of loop extrusion v and independent of other parameters, indicating unobstructed loop extrusion by LEFs. In the dense regime, dynamics slows down drastically and the autocorrelation time exceeds τ, showing that the loop structure persists after multiple rounds of LEF exchange. In the dense regime, the autocorrelation time normalized by τ scales as $\sqrt{\frac{\lambda}{d}}$.

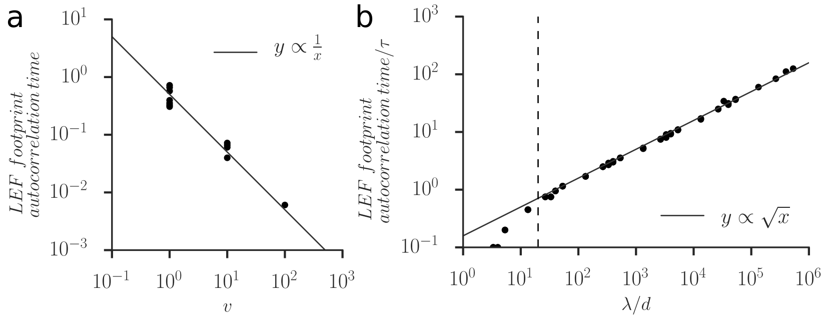

Figure S2: The autocorrelation time of LEF footprints in the sparse (a) and dense (b) regimes. In the sparse regime, we varied N between 10 and 400 LEFs, τ between 10 and 100, and v between 1 and 10. In the dense regime, N was varied between 100 and 1600 LEFs, τ between 10 and 10000, and v between 1 and 100. The vertical dashed line in (b) shows the approximate boundary of the dense regime, $\frac{\lambda}{d} \approx 20$.

2.2 The distribution of loop lengths is exponential in the sparse regime and normal in the dense regime.

The two regimes also have different statistics of loop lengths: in the sparse regime, the loop lengths are distributed exponentially; in the dense regime, the lengths are distributed approximately normally (Fig. S3).

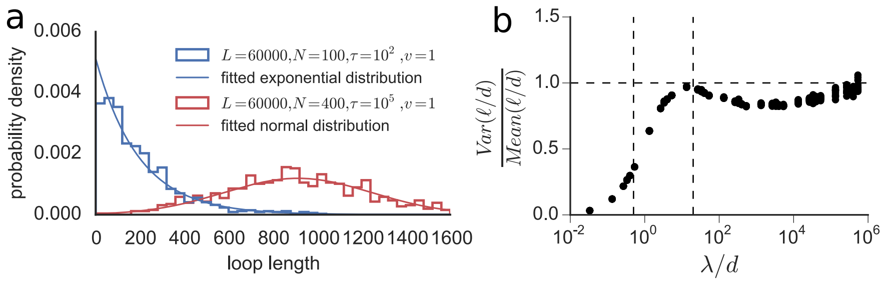

Figure S3: (a) Loop lengths in the sparse and dense regime follow different statistics. In the sparse regime, shown in blue, loop lengths are distributed exponentially; in the dense regime, shown in read, loop lengths are distributed approximately normally. (b) The variance-to-mean ratio of loop lengths normalized by LEF separation d is close to 1.0 in the dense regime.

The exponential distribution of loop lengths in the sparse regime is explained by the simple LEF dynamics. Since LEFs are separated by large gaps, they rarely block each other and extrude loops continuously throughout their residence time on the chromosome. Therefore at every moment of time, the length of a loop is proportional to the amount of time passed since its LEF bound

to the chromosome. In the theory of renewal processes this amount of time is called *age* and, like the residence time of LEF, it is distributed exponentially around its mean of τ [4]. Thus, the lengths of loops in the sparse regime also become exponentially distributed with the average length of $\overline{\ell} = \lambda = 2v\tau$.

In the dense regime, the distribution of loop lengths can be approximated by the normal distribution, with the mean and the standard deviation that depend on the parameters of the system. Interestingly, the variance-to-mean ratio of loop lengths in units of LEF separation d is very close to unity in the dense regime. Our theory presented below explains why the distribution of loop lengths ℓ has a non-zero peak and predicts its approximate location, but it cannot predict the exact analytical form of this distribution nor its width.

2.3 Gaps disappear exponentially with λ/d.

The simple structure of loops in the sparse regime allows us to explain the observed dependence of loop coverage $1 - g$ on $\frac{\lambda}{d}$ (where g is the portion of gaps, i.e. the portion of chromatin fiber that is not extruded into any loop) (Fig. S4). When we increase the number of LEFs N, while keeping L, v and τ fixed, the portion g of gaps should decrease. This dependence is, however, not linear: as more chromatin fiber is extruded into loops, it becomes increasingly likely for LEFs to form nested loops (i.e. bind within already extruded loops) and thus not contribute to the overall loop coverage. Therefore, every new LEF in the system that lands in a gap between loops increases loop coverage by $\frac{\overline{\ell}}{L} \approx \frac{\lambda}{L}$ (i.e. reduces the portion of gaps g by the same amount). The chance of a LEF landing in a gap is g, which gives us a simple differential equation:

$$\frac{dg}{dN} = -\frac{\lambda}{L} g,$$

$$g = e^{-\frac{\lambda}{d}} \tag{S1}$$

Comparison with the simulations (Figure S4) shows that this solution captures the transition between the sparse and the dense regimes, but noticeably underestimates the portion of gaps as we approach the dense regime, $1 < \frac{\lambda}{d} < 20$. This discrepancy is due to the growing difference between $\overline{\ell}$ and λ in the dense regime (see below).

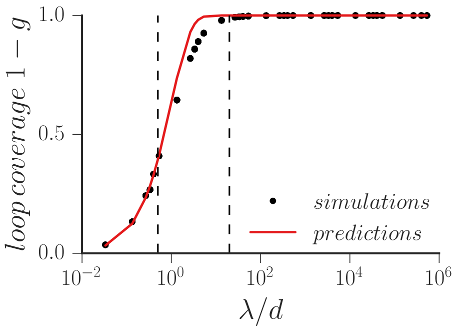

Figure S4: The comparison of the theoretically predicted loop coverage with the results of the simulations. The vertical dashed lines show the approximate boundaries of the sparse and dense regimes.

3 The theory of self-organization of loop arrays in the dense regime.

3.1 In the dense regime, loops have stable lengths and fluctuating numbers of reinforcing LEFs.

At $\lambda \gg d$, gaps between loops disappear and the system transitions into the *dense* regime. The condition $\lambda \gg d$ means that LEFs can potentially form loops that are much larger than the amount of chromatin available to each of them, so the size of the extruded loop become limited by collisions between LEFs. Also, because there are no gaps between loops, LEFs that rebind to the chromatin always start new loops within other already existing loops. Finally, as we show in the main text, in the extreme dense regime loop branching becomes increasingly rare and the majority of LEFs just stack on top of each other, forming reinforced loops. Thus, LEFs in the dense regime fold chromatin into an array of consecutive reinforced loops. The length ℓ of each reinforced loop is relatively stable: supported by multiple LEFs, it does not disappear or shrink, when some of them unbind, but it cannot grow either, because its LEFs are blocked by the neighbors. Conversely, the number of LEFs n in each reinforced loop constantly fluctuates: the loops constantly lose LEFs due to their unbinding and receive new LEFs that rebind to the chromosome from the solution. Below we describe the fluctuations of loop structure in the dense regime and show how they lead to a globally stable steady state.

3.2 The number of LEFs in a loop is approximately Poisson-distributed around ℓ/d.

Let us derive the distribution of the number of LEFs n supporting a single reinforced loop of length ℓ. The loop loses LEFs due to their dissociation from

the chromatin fiber. Since individual LEFs unbind with a rate $\mu = \frac{1}{\tau}$, the overall rate of LEF loss depends on n and equals

$$\mu_n = n\mu = \frac{n}{\tau} \tag{S2}$$

The loop also receives a flux of incoming LEFs that bind back to the chromosome from the solution. In our simple model, LEFs rebind to random sites and thus have a chance $\frac{\ell}{L}$ of landing within the chosen loop. As a result, the body of the loop serves as an antenna: the larger the loop is, the more LEFs it receives. At every moment of time, LEFs unbind and immediately rebind to the chromosome with the rate $N \cdot \frac{1}{\tau}$, so that the rate r of LEF binding to the chosen loop equals

$$r = \frac{N}{\tau} \cdot \frac{\ell}{L} = \frac{1}{\tau}\frac{\ell}{d} \tag{S3}$$

This stochastic gain and loss of LEFs produces fluctuations of the number of LEFs in the loop. The equations S2 and S3 allow us to find the probability p_n for the loop to have n LEFs. In a population of loops of equal length ℓ, the fraction p_n of loops supported by n LEFs changes over time because of three factors: a) these loops gain or lose LEFs with a combined rate $r + \mu_n$, b) loops with $n-1$ LEFs gain LEFs at rate r and c) loops with $n+1$ LEF lose LEFs at rate μ_{n+1}. The dynamics of the fraction p_n is then described with:

$$\begin{cases} \frac{dp_n}{dt} = -\left(r + \mu_n\right) p_n + r p_{n-1} + \mu_{n+1} p_{n+1}, & n > 1 \\ \frac{dp_1}{dt} = -\left(r + \mu_1\right) p_1 + \mu_2 p_2, & n = 1 \end{cases}$$

The loss of the last LEF is irreversible and loops without LEFs disappear. In order to find a quasi steady state distribution of p_n described by $\frac{dp_n}{dt} = 0$, we have to ignore this fact for now and set $\mu_1 = 0$. This gives us the following system of equations [5]:

$$\begin{cases} (r + \mu_n) p_n = r p_{n-1} + \mu_{n+1} p_{n+1}, & n > 1 \\ r p_1 = \mu_2 p_2 \end{cases}$$

The solution of this system shows that the number of LEFs n, $n \geq 1$ in a loop is Poisson-distributed:

$$p_n = \prod_{i=1}^{n-1} \frac{r_i}{\mu_{i+1}} p_1 = \frac{1}{n!}\left(\frac{r}{\mu}\right)^n \frac{1}{e^{\frac{r}{\mu}} - 1}$$

We can relate this distribution to the size of the loop and density of LEFs using the expressions (S3) and (S2):

$$p_n\left(\ell/d\right) = \frac{1}{n!}\left(\ell/d\right)^n \frac{1}{e^{\ell/d} - 1} \tag{S4}$$

The average number of LEFs $\overline{n}$ in a loop then equals:

$$\bar{n} = \frac{\ell}{d}\frac{1}{1 - e^{-\ell/d}} \tag{S5}$$

This expression highlights the importance of the length scale d: the length of a loop expressed in units of LEF separation d is approximately equal to the average number of LEFs in this loop. Below we will often use the loop length normalized by d:

$$\ell_d \equiv \frac{\ell}{d} \tag{S6}$$

such that:

$$\overline{n} \approx \ell_d \tag{S7}$$

3.3 The lifespan of reinforced loops increases exponentially with their length.

The derived distribution (S4) of the number of LEFs per loop allows us to estimate the average lifespan of a loop $\bar{t}$. Loops get disassembled when they lose their last LEF. Therefore, the rate of loop death can be estimated as:

$$R_{death} \approx \mu p_1 = \frac{1}{\tau}\frac{\ell_d}{e^{\ell_d} - 1} \approx \frac{1}{\tau}\ell_d e^{-\ell_d} \tag{S8}$$

And the average lifespan $\bar{t}$ of a loop of length ℓ_d is

$$\bar{t} = \frac{1}{R_{death}} \approx \tau\frac{e^{\ell_d} - 1}{\ell_d} \tag{S9}$$

The asymptotic dependence of $\bar{t}$ on the length of the normalized loop length ℓ_d is then:

$$\bar{t}_{asymp} = \frac{1}{R_{death}^{asymp}} \approx \tau\frac{e^{\ell_d}}{\ell_d} \tag{S10}$$

The equation (S10) reveals that the lifespan of a loop grows almost exponentially with its length. Thus, reinforcement makes longer loops essentially immortal: for example, loops with length $\ell_d = 6.5$ live 100 times longer than individual LEFs, and those with $\ell_d = 9$ live 1000 times longer. However, the effects of reinforcement are significant only for longer loops ($\ell_d \gtrsim 3.5$) with the lifespan extension to 10τ and more.

The simple functional form of Eq. (S10) allows us to use it in analytical calculations. However, in its derivation we assumed that loop death does not perturb the distribution of n, which is not necessarily true. In chapter (4.1), we will derive the expression for $\bar{t}$ without this assumption and show that our estimates (S9) and (S10) are, in fact, very accurate.

3.4 Loops in the dense regime stochastically divide in two.

Loops in the dense regime divide when two LEFs land within the same loop and extrude two separate loops instead of stacking on top of one another. Both daughter loops then start receiving reinforcing LEFs, thus, cutting the supplies off the mother loop. This causes the mother loop to disassemble on the timescale of several τ (for more accurate estimate, see Chapter (4.4)) and the two daughter loops take its place. This process creates new loops on the chromatin in the dense regime.

We can estimate how the rate of loop division depends on the parameters of the system. LEFs land into a loop of size ℓ with a rate of $\frac{N\ell}{\tau L} = \frac{1}{\tau}\frac{\ell}{d}$. A loop divides when the second LEF lands before the first LEF has fully expanded to the loop borders, within a time window of $\sim \frac{\ell}{2v}$. Thus, the rate of loop division scales as:

$$R_{div} \sim \left(\frac{1}{\tau}\frac{\ell}{d}\right)^2 \frac{\ell}{2v} = \frac{1}{\tau}\ell_d^3 \frac{d}{\lambda} \tag{S11}$$

However, these two LEFs can still stack on one another. In fact, as the first LEF extrudes a bigger and bigger loop, there is less chance for the second LEF to land outside of its loop. Thus, a more accurate general expression for R_{div} should look like:

$$R_{div} = r^2 \int_0^\infty P_{div}(\ell, t)\, dt, \tag{S12}$$

where $P_{div}(\ell, t)$ is the probability for two LEFs to divide a loop of length ℓ if they land with a time delay t.

The loop splits if the second LEF lands outside of the loop extruded by the first. Integrating over all possible positions of the two LEFs, x_1 and x_2, we get:

$$\begin{aligned} P_{div}(\ell, t) &= \frac{1}{l^2}\int_0^l dx_1 \int_0^l dx_2 \left(\mathbf{1}_{x_2 > x_1 + vt} + \mathbf{1}_{x_2 < x_1 - vt}\right) = \\ &= \left(1 - \frac{vt}{\ell}\right)^2 \end{aligned} \tag{S13}$$

Here, $\mathbf{1}_{condition}$ is the indicator function, which equals 1 is the condition is true and 0 otherwise. The expression for R_{div} is then:

$$R_{div}\left(\ell_d, \frac{\lambda}{d}\right) = r^2 \int_0^{\ell/v} P_{div}(\ell, t)\, dt = \frac{2}{3}\frac{1}{\tau}\ell_d^3 \frac{d}{\lambda} \tag{S14}$$

In other words, stacking of LEFs decreases the rate of loop division by $^2/_3$.

3.5 The balance between loop death and division gives rise to the steady state of the dense regime.

In the steady state of the dense regime, the average length $\bar{\ell}$ of all loops in the system stays approximately constant over time. This implies that the number

of loops on the chromosome is also constant, therefore, the global rate of loop creation through division should be equal the global rate of loop death. Assuming that all loops in the system have the same length $\ell = d\bar{\ell_d}$, we get the steady state condition:

$$R_{div}\left(\bar{\ell}_d, \frac{\lambda}{d}\right) = R_{death}\left(\bar{\ell}_d\right) \tag{S15}$$

Plugging in the previously obtained estimates for R_{death} (S8) and R_{div} (S14), we get:

$$\frac{2}{3}\frac{1}{\tau}\ell_d^3\frac{d}{\lambda} = \frac{1}{\tau}\ell_d e^{-\ell_d}$$

The solution of this equation defines us the average length and the number of LEFs per loop in the steady state:

$$\bar{\ell} = 2dW\left(\sqrt{\frac{3\lambda}{8d}}\right) \tag{S16}$$

$$\bar{n} \approx \ell_d = 2W\left(\sqrt{\frac{3\lambda}{8d}}\right) \tag{S17}$$

Here, $W(x)$ is the Lambert W function, defined as the solution of $W(x)e^{W(x)} = x$. For $\frac{\lambda}{d}$ varying between 20 and 10^6, we can use the approximation $W(x) \approx 0.3 + \ln(x) - \ln(\ln(x))$:

$$\bar{\ell} \approx d\left[0.3 + \ln\left(\frac{3\lambda}{8d}\right) - \ln\ln\left(\frac{3\lambda}{8d}\right)\right] \tag{S18}$$

$$\bar{n} \approx 0.3 + \ln\left(\frac{3\lambda}{8d}\right) - \ln\ln\left(\frac{3\lambda}{8d}\right) \tag{S19}$$

Our theory also explains why the steady state is globally stable. The average steady state loop length $\bar{\ell}$ (S16) is located at the intersection of the curves $R_{death}(\ell, d)$ (S10) and division $R_{div}(\ell, \lambda, d)$ (S14) (Fig. S5); loops longer than $\bar{\ell}$ are more likely to divide into smaller loops, loops shorter than $\bar{\ell}$ are more likely to die and let their neighbors grow. Thus, the scalings of $R_{death}(\ell, d)$ (S10) and division $R_{div}(\ell, \lambda, d)$ (S14) focus the distribution of the loop sizes around the average value $\bar{\ell}$, which explains the approximately normal distribution of loop sizes shown on Fig.(S3).

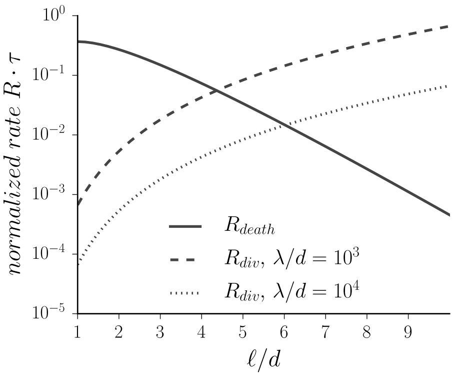

Figure S5: The scalings of R_{death} and R_{div} with the loop length ℓ. The average loop length at the steady state is located at the intersection of the two curves. The difference in the derivative signs of R_{death} and R_{div} provides global stability of the steady state.

The equation (S16) is approximate and thus can be not accurate enough. For the applications requiring ~1% precision, we used a 7-th degree polynomial expression which was fit to $\log_{10} \bar{\ell}(\frac{\lambda}{d})$ observed in the simulations with $\frac{\lambda}{d}$ in the range $(10^{-1.5}; 10^{5.5})$:

$$\log_{10}\left(\frac{\bar{\ell}}{d}\right) = -0.08238 + 0.7258x - 0.2514x^2 - 0.003995x^3 + 0.03445x^4 - 0.01077x^5 + 0.001371x^6 - 6.472 \cdot 10^{-5}x^7, \; x = \log_{10}\left(\frac{\lambda}{d}\right)$$

3.6 Maximal lengthwise compaction is achieved on the lower border of the dense regime.

LEFs fold a chromosome into a system of consecutive loops and thus dramatically reduce its length (Fig. S6). A biologically important question is what amount of LEFs would maximize lengthwise compaction of the chromosome, given their microscopic properties.

The length of a chromosome compacted by LEFs, L_{comp}, has two components: (a) the combined widths of loop bases and (b) the length of gaps between loops (Fig. S6). A loop base has a width of *at least* the thickness of chromatin fiber, a (in our model, it equals 1 site or ~10 nm), so that the minimal estimate for L_{comp} is:

$$L_{comp} = a n_{loops} + gL = a\frac{L}{\bar{\ell}} + gL, \tag{S20}$$

The coefficient of lengthwise compaction is then given by

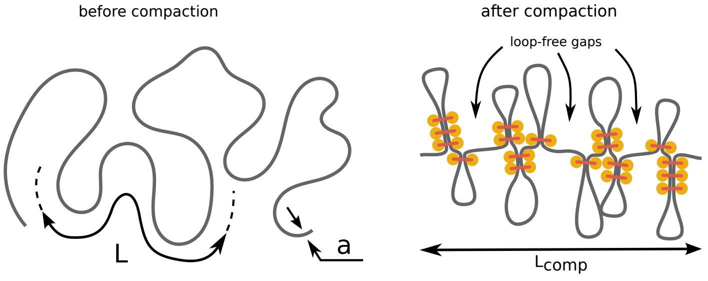

Figure S6: LEFs drastically reduce the length of a chromosome by folding it into a system of consecutive loops. The length of a compacted chromosome is determined by the combined length of the loop bases and the length of gaps between loops.

$$c_{len} = \frac{L}{L_{comp}} = \frac{1}{{}^{a}/_{\bar{\ell}} + g} \tag{S21}$$

The portion of loop-free gaps g decreases almost exponentially with the number of LEFs as the system approaches the dense regime (see Section 2.3). On contrary, the combined length of loop bases is proportional to the number of loops in the system and grows almost linearly with the number of LEFs. Therefore, maximal compaction is achieved at the lower boundary of the dense regime, where the gaps disappear, but the number of loops is still low. Simulations confirm our reasoning (Fig.S7), but show that the exact location of the optimum depends slightly on λ. This dependence is caused by the presence of exponentially small residual gaps that prevent the system from achieving extremely high degrees of compaction.

3.7 The maximal degree of total compaction depends on the length of the chromosome and the size of LEFs.

The extreme values of the coefficient of lengthwise compaction can be misleading: high lengthwise compaction is achieved when the chromosome is folded into a few very large loops, so that the width of the compacted structure can be larger than its length. Formally, the maximal lengthwise compaction is achieved when the whole chromosome is folded into one loop! A more meaningful measure of compaction is the ratio of the chromosome length L to the maximum of its width and length, or, the coefficient of total compaction c_{tot}. Since polymers folded into loop arrays naturally assume bottle-brush conformations [6] with loops extending away from the backbone formed by loop bases, we set the width of a compacted chromatin fiber to be roughly the size of individual loops $\bar{\ell}$:

$$c_{tot} = min\left(\frac{L}{L_{comp}}, \frac{L}{\bar{\ell}}\right)$$

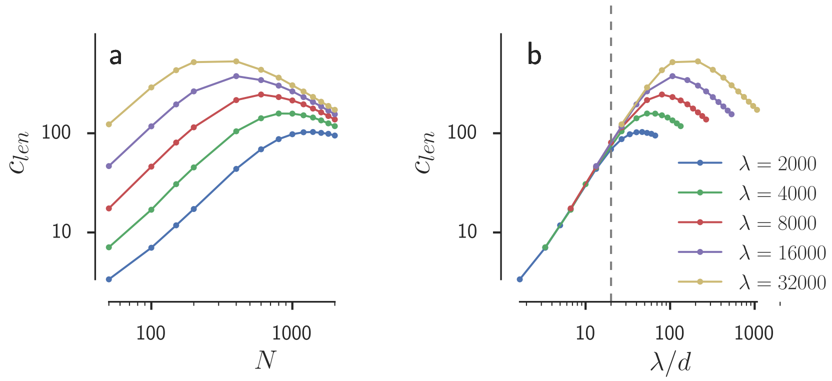

Figure S7: (a) Given the microscopic properties of LEFs, there is an optimal amount of LEFs that provides maximal lengthwise compaction. (b) Maximal lengthwise compaction occurs at the lower boundary of the dense regime where the gaps disappear, but the number of loops is relatively low.

Then the maximal total compaction is achieved when the width and length are equal:

$$\bar{\ell}^* = L_{comp} = a\frac{L}{\bar{\ell}^*} + gL$$

Ignoring the contribution of gaps, we can obtain the expression for the optimal loop length:

$$\bar{\ell}^* = \sqrt{aL}$$

The maximal degree of total compaction is then:

$$c^*_{tot} = \frac{L}{\bar{\ell}^*} = \sqrt{\frac{L}{a}} \tag{S22}$$

Our simulations confirm the existence of a global maximum of c_{tot} expressed by Eq. S22 (Fig. S8). The number of LEFs required to reach this maximum almost does not depend on λ. This is not surprising given that the average loop length in the steady state (Eq. (S16)) depends linearly on N and only logarithmically on λ. However, in order to reach the maximal total compaction, LEFs must have sufficiently large λ, otherwise they can not generate enough lengthwise compaction (S7).

3.8 Gradual activation of LEFs speeds up convergence to the steady state.

In our simulations, the steady state was achieved on long timescales, up to $10^3\tau$. With the experimental estimates of τ of at least a few minutes [7], cells

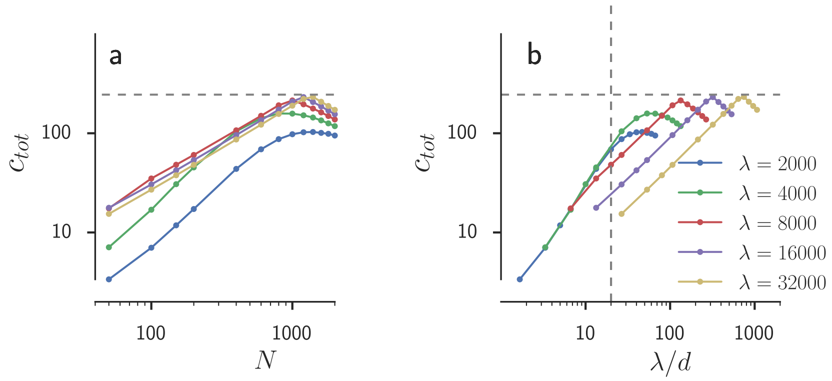

Figure S8: (a) The maximal total compaction and the number of LEFs required to achieve it almost does not depend on λ. (b) As λ increases, the maximal total compaction is achieved at higher values of $\frac{\lambda}{d}$. The horizontal dashed lines show the predicted maximal coefficient of total compaction $c^*_{tot} = \sqrt{L/a}$; the vertical dashed line shows the lower boundary of the dense regime $\lambda/d \approx 20$.

might not have enough time to compact their chromosomes using the mechanism described above. Below we show that gradual activation of LEFs reduces the timescales of convergence to the steady state below τ, thus allowing fast chromosome compaction in mitosis.

The transition to the steady regime in our simulations is slow because we initiate the system far from the steady state. When we activate all LEFs simultaneously, they initially fold the chromosome into an array of small loops; some of these loops then slowly die and the remaining loops grow, until the average size of a loop reaches the steady state value. We can achieve a faster approach to the steady state if by initial activation of a small fraction of LEFs or if by their gradual activation.

By choosing a proper number of LEFs to activate, we can adjust the length of the initially extruded loops to be equal the steady state length. After initial loops are formed, we activate the rest of the LEFs. In this scenario the system will rapidly achieve the steady state because this second batch of LEFs will reinforce the loops formed by the first batch. The required number of initially activated LEFs is then given by the condition $\frac{L}{N_{init}} = \bar{\ell}$:

$$N_{init} = \frac{L}{\bar{\ell}}$$

These LEFs would form an array of consecutive loops over the period of time $t_{init} \sim \frac{\bar{\ell}}{2v}$, after which we could add the rest of the LEFs.

The same result can be achieved using a more realistic scenario where all LEFs are gradually (stochastically) activated with the activation rate $1/t_a$. The number of active LEFs in a system at time t then equals:

$$N(t) = N\left(1 - e^{-t/t_a}\right)$$

The optimal activation period t_a^{opt} can be found by comparing this approach with the two-step activation scheme, so that by the time t_{init} the system has N_{init} active LEFs:

$$N\left(1 - e^{-\frac{t_{init}}{t_a^{opt}}}\right) = N_{init}$$

which gives us the following expression for optimal activation time t_a^{opt}:

$$t_a^{opt} = \tau\frac{\bar{\ell}^2}{d\lambda} = \tau\frac{d}{\lambda}\bar{\ell}_d^2 \tag{S23}$$

In order to confirm Eq. (S23), we simulated stochastic activation of LEFs in a wide range of parameters and found the optimal LEF activation times t_a^{opt}. These values of t_a provided the fastest convergence to the steady state, as measured by the root mean square deviation of the loop length trajectory $\bar{\ell}(t)$ from the steady state value. The simulations showed that the expression (S23) captured the major mechanism behind the optimal LEF activation (Fig. S9).

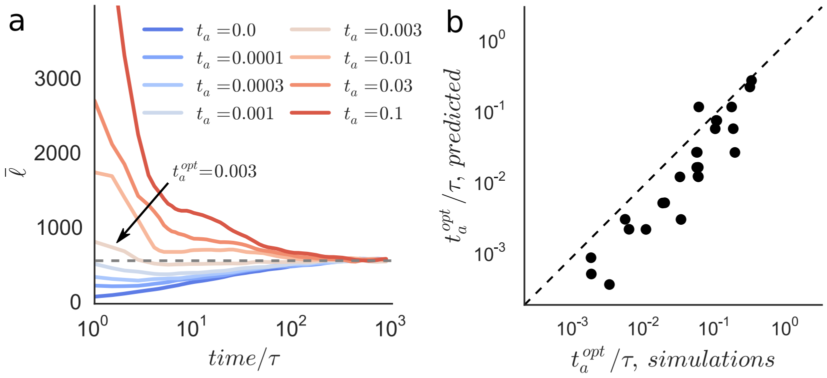

Figure S9: (a) Stochastic activation of LEFs with a rate $1/t_a$ speeds up convergence of the average loop length $\bar{\ell}$ to its steady state value (the gray dashed line). The optimal activation delay t_a^{opt} minimizes the root mean square deviation of the curve $\bar{\ell}(t)$ from the steady state value. (b) The values of t_a^{opt} predicted with Eq. (S23) agree reasonably well with the optimal LEF activation times found in simulations across a wide range of system parameters.

The systematic discrepancy between the optimal activation times in simulations and those predicted by Eq. (S23) is due to two major factors:

a) we assumed that the LEFs activated after formation of a loop array serve only to reinforce the existing loops. However, these LEFs also divide the existing loops via the mechanism described in Chapter 3.4 and thus increase the number of created loops.

b) Some portion of the initially established $N_{initial}$ loops dies before a loop array get established, thus decreasing the number of created loops.

Finally, it is important to note that we only considered convergence of the mean loop length $\bar{\ell}$; convergence of the other moments of the distribution of loop length might take a different amount of time.

4 Corrections to the theory of self-organization of loop arrays.

4.1 The theory of stochastic processes provides an exact expression for the lifespan of loops.

The estimate of the rate of loop death (S8) involved several approximations and thus can potentially be inaccurate. In the next four chapters 4.1-4.4, we will use the theory of stochastic processes to derive an accurate model of loop death and estimate the precision of (S8).

We model the fluctuations of the number of LEFs n supporting a loop with the stochastic immigration-death process: the loop receives a steady flux of incoming LEFs (immigration), but each of them lives for a finite period of time (death). This process has been extensively studied in the literature [5] and we adapt the existing derivations to our model.

In the language of immigration-death processes, the death of loops upon loss of all LEFs corresponds to an adsorbing state at $n = 0$. Then the average lifespan of a loop is defined as the mean time to absorption into the state $n = 0$ and depends on the initial number of LEFs n. The lifespans $\bar{t}_n$ for different n are related to each other: a loop with n LEFs keeps the same n for an average period of $\frac{1}{r+\mu_n}$ and then either receives an extra LEF with a probability $\frac{r}{r+\mu_n}$ and lives for another period $\bar{t}_{n+1}$ or loses a LEF with a probability $\frac{\mu_n}{r+\mu_n}$ and lives for $\bar{t}_{n-1}$ (μ_n and r were defined in Eq.(S2) and Eq.(S3)). This allows us to relate different $\bar{t}_n$ with a system of equations:

$$\begin{cases} \bar{t}_n = \frac{1}{r+\mu_n} + \frac{r}{r+\mu_n}\bar{t}_{n+1} + \frac{\mu_n}{r+\mu_n}\bar{t}_{n-1}, \quad n > 0 \\ \bar{t}_0 = \frac{1}{r+\mu_0} + \frac{r}{r+\mu_0}\bar{t}_1 \end{cases} \tag{S24}$$

This system can be converted into a recursive equation using a new variable $\delta\bar{t}_n$,

$$\delta\bar{t}_n = \bar{t}_n - \bar{t}_{n+1},$$

such that $\delta\bar{t}_0 = -\bar{t}_1$ and $\bar{t}_n = -\sum_{i=0}^{n-1} \delta\bar{t}_i$. Plugging $\delta\bar{t}_n$ into the system of equations (S25), we obtain a recursive relation

$$\delta\bar{t}_n = \frac{1}{r} + \frac{\mu_n}{r}\delta\bar{t}_{n-1}$$

Then,

$$\begin{aligned} \delta\bar{t}_1 &= \frac{1}{r} + \frac{\mu_1}{r}\delta\bar{t}_0 = \frac{1}{r} - \frac{\mu_1}{r}\bar{t}_1 \\ \delta\bar{t}_2 &= \frac{1}{r} + \frac{\mu_2}{r}\delta\bar{t}_0 = \frac{1}{r} + \frac{\mu_2}{r^2} - \frac{\mu_1\mu_2}{r^2}\bar{t}_1 \\ &\dots \\ \delta\bar{t}_n &= \sum_{i=1}^{n} \frac{1}{r} \prod_{j=i+1}^{n} \frac{\mu_j}{r} - \left(\prod_{i=1}^{n} \frac{\mu_i}{r}\right)\bar{t}_1 \end{aligned}$$

Defining an auxiliary variable ρ_n, we get:

$$\rho_n = \frac{\mu_1 \ldots \mu_n}{r_1 \ldots r_n} = \frac{n!}{\ell_d^n}$$

$$\delta \bar{t}_n = \sum_{i=1}^{n} \frac{1}{r} \frac{\rho_n}{\rho_i} - \rho_n \bar{t}_1 = \rho_n \left(\sum_{i=1}^{n} \frac{1}{r\rho_i} - \bar{t}_1 \right)$$

$$\bar{t}_1 = \sum_{i=1}^{\infty} \frac{1}{r\rho_i} + \lim_{n\to\infty} \frac{1}{\rho_n} \left(\bar{t}_n - \bar{t}_{n+1} \right)$$

The second term is zero because $\lim_{n\to\infty} \frac{e^n}{n!} = 0$, therefore

$$\bar{t}_1 = \sum_{i=1}^{\infty} \frac{1}{r\rho_i}$$

This finally allows us to find $\bar{t}_n$:

$$\begin{aligned}
\bar{t}_n &= -\sum_{i=0}^{n-1} \delta \bar{t}_i = \bar{t}_1 - \sum_{i=1}^{n-1} \delta \bar{t}_i = \\
&= \bar{t}_1 - \sum_{i=1}^{n-1} \rho_i \left(\sum_{j=1}^{i} \frac{1}{r\rho_j} - t_1 \right) = \bar{t}_1 - \sum_{i=1}^{n-1} \rho_i \left(\sum_{j=1}^{i} \frac{1}{r\rho_j} - \sum_{j=1}^{\infty} \frac{1}{r\rho_j} \right) \\
&= \bar{t}_1 + \sum_{i=1}^{n-1} \rho_i \sum_{j=i+1}^{\infty} \frac{1}{r\rho_j} = \\
&= \sum_{i=1}^{\infty} \frac{1}{r\rho_i} + \sum_{i=1}^{n-1} \rho_i \sum_{j=i+1}^{\infty} \frac{1}{r\rho_j} = \\
&= \frac{1}{r} \left[\sum_{i=1}^{\infty} \frac{1}{i!} \left(\frac{r}{\mu} \right)^i + \sum_{i=1}^{n-1} i! \left(\frac{\mu}{r} \right)^i \sum_{j=k+1}^{\infty} \frac{1}{j!} \left(\frac{r}{\mu} \right)^j \right] = \\
&= \frac{1}{r} \left[e^{\frac{r}{\mu}} - 1 + \sum_{i=1}^{n-1} \sum_{j=1}^{\infty} \frac{i!}{(i+j)!} \left(\frac{r}{\mu} \right)^j \right] = \\
&= \frac{1}{r} \left[e^{\frac{r}{\mu}} - 1 + e^{\frac{r}{\mu}} \sum_{i=1}^{n-1} \left(\frac{\mu}{r} \right)^i \left(\Gamma(i+1) - \Gamma(i+1, \frac{r}{\mu}) \right) \right]
\end{aligned}$$

$$\bar{t}_n(\ell_d) = \frac{\tau}{\ell_d} \left[e^{\ell_d} - 1 + e^{\ell_d} \sum_{i=1}^{n-1} \frac{i! - \Gamma(i+1, \ell_d)}{\ell_d^i} \right] \tag{S25}$$

where $\Gamma(a, x)$ is the upper incomplete Gamma function. Interestingly, the previously obtained expression for $\bar{t}$ (S9), in fact, equals the average lifespan of a loop with a single LEF:

$$\bar{t}_1(\ell_d) = \tau \frac{e^{\ell_d} - 1}{\ell_d} \tag{S26}$$

The expression (S25) is too bulky to be used in analytical derivations or provide any new intuitive understanding. Below we show that most of the terms in (S25) in fact can be dropped to obtain a compact, yet accurate approximation of $\bar{t}_n$.

4.2 Loops supported by a single LEF can die immediately.

The Eq. (S25) gives us the average loop lifespan, but does not tell anything about the distribution of t_n. In order to find it, we simulated 10^5 stochastic immigration-death processes with $\overline{n} = 3$ with $n_0 = 1$ and $n_0 = 3$ initial LEFs (Fig. (S10)). Both distributions had exponential tails, but the distribution of $\bar{t}_1$ had an additional peak around zero (Figure S10). The normalized tails of the distributions for the two initial conditions perfectly matched at $t_n \geq 3\tau$, indicating that the starting number of LEFs did not affect the later stages of loop dynamics.

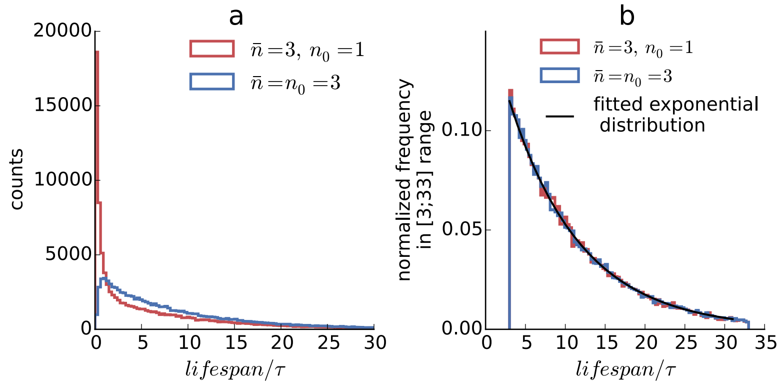

Figure S10: The distribution of the lifespans of immigration-death processes with different initial conditions. (a) The overall distribution; (b) the normalized lifespan frequency for the processes that survived the initial period $t_n < 3\tau$.

The increased frequency of short lifespans of single-LEF loops is caused by stochastic fluctuations: with some probability these loops immediately lose their only LEF and disassemble before receiving any reinforcing LEFs. A rough estimate for the probability of such immediate death is:

$$p_1^{death}(\ell_d) \approx \frac{\mu}{r + \mu} = \frac{1}{\ell_d + 1} \tag{S27}$$

We can find a more accurate expression for p^{death} using the theory of immigration-death processes. If we define immediate death more generally as an event when a

loop dies before reaching its fully reinforced level $n = \overline{n}$, we get an immigration-death process with two adsorbing boundaries at $n = 0$ and $n = \overline{n}$. Then the probability p_i^{death} that a loop with i LEFs will die before accumulating $\overline{n}$ LEFs, obeys the following system of equations:

$$\begin{aligned}
p_0^{death} &= 1 \\
&\cdots \\
p_i^{death} &= \frac{\mu_i}{r+\mu_i} p_{i-1}^{death} + \frac{r}{r+\mu_i} p_{i+1}^{death} \\
&\cdots \\
p_{\overline{n}}^{death} &= 0
\end{aligned}$$

And the solution is:

$$p_i^{death} = \frac{\sum_{k=i}^{\overline{n}-1} k! \left(\frac{\mu}{r}\right)^k}{\sum_{k=0}^{\overline{n}-1} k! \left(\frac{\mu}{r}\right)^k}$$

Particularly, a loop formed by a single LEF dies before accumulating $\overline{n}$ LEFs with the probability

$$p_1^{death}(\ell_d) = 1 - \left[\sum_{k=0}^{l_d-1} \frac{k!}{(\ell_d)^k}\right]^{-1} \tag{S28}$$

4.3 Accounting for immediate death gives a simple and accurate estimate for the rate of loop death.

The analysis above suggests two different processes lead to loop death: (a) immediate death before full reinforcement, which probability depends on the initial number of LEFs n_0, (b) exponential death of fully reinforced loops, which rate is independent of n_0. This allows us to find a compact estimate for $\bar{t}_n(\ell_d)$. Roughly, a single-LEF loop either dies immediately with a chance p_1^{death} or quickly accumulates $\overline{n}$ LEFs and lives for $\bar{t}_{\overline{n}}$:

$$\bar{t}_1(\ell_d) \approx p_1^{death}(\ell_d) \cdot 0 + \left(1 - p_1^{death}(\ell_d)\right) \bar{t}_{\overline{n}}(\ell_d)$$

Then the lifespan of a loop with $n_0 = \overline{n}$ LEFs is given by:

$$\bar{t}_{\overline{n}}(\ell_d) \approx \frac{\bar{t}_1(\ell_d)}{1 - p_1^{death}(\ell_d)} = \frac{\tau}{\ell_d}\left(e^{\ell_d} - 1\right) \sum_{k=0}^{\ell_d-1} \frac{k!}{\ell_d^k} \tag{S29}$$

This formula can be generalized for an arbitrary initial number of LEFs n_0, $1 \leq n_0 \leq \overline{n}$:

$$\bar{t}_{n_0}(\ell_d) \approx \frac{1 - p_{n_0}^{death}(\ell_d)}{1 - p_1^{death}(\ell_d)} \bar{t}_1(\ell_d) \tag{S30}$$

Now we can compare the accuracy of the expressions (S25), (S10) and (S29). We simulated the dynamics of LEF number n using a stochastic immigration-death process with different average numbers of LEFs $\overline{n}$, $n_0 = \overline{n}$. Simulations at each value of $\overline{n}$ were repeated 1000 times to obtain enough statistics (Figure (S11)).

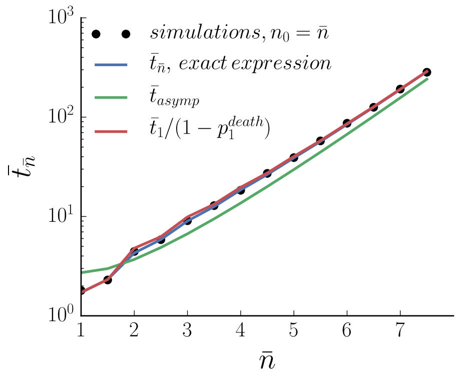

Figure S11: The average lifespan of a loop can be predicted accurately with analytical expressions. The black dots show the results of simulations, the predictions with analytical expressions (S25), (S10) and (S29) are shown in blue, green and red, correspondingly.

The simulations show that the asymptotic expression (S10) significantly overestimates the lifespan $\bar{t}$ of short loops ($\ell_d < 2$) and underestimates $\bar{t}_{\bar{n}}$ for longer loops. On contrary, the approximate expression (S29) is as accurate as the exact expression (S25) and thus can be used instead.

4.4 The lifespan of a loop after interruption of reinforcement is approximately Gumbel-distributed.

In our theory of loop division, we assumed that "mother" loops die soon after interruption of reinforcement. In this chapter we support this assumption with analytical derivations and obtain the full distribution of residual lifetimes of loops.

A loop exists as long as it has at least one LEF at its base. Therefore, its residual lifespan t_{res} after interruption of reinforcements is given by the maximum of the residence times of its n_0 LEFs. This allows us to write the cumulative distribution function (CDF) of t_{res} as the CDF of the maximum of n_0 exponentially distributed lifetimes of individual LEFs:

$$F_{n_0}(t_{res}) = F\{max(t_1, .., t_{n_0}) \leq t_{res}\} = \left[1 - \exp\left(-\frac{t_{res}}{\tau}\right)\right]^{n_0}$$

The probability density function of t_{res} is then given by:

$$f_{n_0}(t_{res}) = \frac{d}{dt_{res}} F\{max(t_1, .., t_{n_0}) \le t_{res}\} = \frac{1}{\tau} n_0 \left[1 - \exp\left(-\frac{t_{res}}{\tau}\right)\right]^{n_0 - 1} \exp\left(-\frac{t_{res}}{\tau}\right)$$

We can rephrase this expression in terms of the known loop length ℓ_d by summing over all possible LEF number n_0:

$$f_{\ell_d}(t_{res}) dt_{res} = \frac{1}{\tau} \sum_{n_0=1}^{\infty} p_{n_0}(\ell_d) f_{n_0}(t_{res}) dt_{res}$$

Since the number of LEFs n_0 is roughly Poisson-distributed around $\overline{n}$ (Eq. (S4)), we get

$$f_{\overline{n}}(t') dt' = e^{\left(-t' - e^{-t'}\right)} dt', \text{where } t' = \frac{t_{res}}{\tau} - \ln \ell_d \tag{S31}$$

The average lifespan of a loop $\bar{t}_{res}$ after interruption of LEFs is then

$$\bar{t}_{res} = \tau \left[\ln \ell_d + \gamma - Ei(-\ell_d) + e^{-\ell_d} \ln \ell_d\right]$$

Here, $\gamma \approx 0.577\ldots$ is the Euler–Mascheroni constant and $Ei(x)$ is the exponential integral. At larger values of ℓ_d this expression can be further simplified to:

$$\bar{t}_{res} \approx \tau \left(\ln \ell_d + \gamma\right) \tag{S32}$$

The expression (S31) looks exactly like the Gumbel distribution, with the only difference that it is defined on $t' \in [-\ln \ell_d; \infty)$, while the Gumbel distribution is defined on the whole real line. The fact that the lifespan of a stack of LEFs is roughly Gumbel-distributed is not surprising: the residual lifespan of a loop equals the maximum of n_0 exponentially-distributed lifespans of individual LEFs and the Gumbel distribution is the limiting distribution of a maximum of n exponential numbers when $n \to \infty$.

4.5 Selection for the minimal daughter loop size slows down loop division.

In the next three chapters 4.5-4.7, we will analyze the inaccuracies of our simple model of loop division and will come up with better estimates for R_{div}.

The first major correction to Eq. (S14) is due to the fact that both daughter loops must be large enough to survive and receive stable reinforcements. A more accurate estimate of R_{div} therefore must discard the configurations of daughter loops where one of them is too small to be viable. The condition on the minimal size of the loop then can be plugged into the expression for the probability of division P_{div} (S13):

$$P_{div}^{minlen}(\ell, t) = \frac{1}{\ell^2} \int_0^{\ell} dx_1 \int_0^{\ell} dx_2 \left(\mathbf{1}_{x_2 > x_1 + vt} + \mathbf{1}_{x_2 < x_1 - vt}\right) \mathbf{1}_{\ell_1 > \ell_0} \mathbf{1}_{\ell_2 > \ell_0}$$

Here, ℓ_0 is the minimal length of a daughter loop, ℓ_1 and ℓ_2 are the lengths of the fully-extruded daughter loops, defined as:

$$\begin{cases} \ell_1 = \frac{x_2+x_1+vt}{2}, & x_1 < x_2 \\ \ell_1 = \frac{x_2+x_1-vt}{2}, & x_1 > x_2 \end{cases} \tag{S33}$$

$$\ell_2 = \ell - \ell_1$$

This gives us the following expression for the rate of loop division:

$$R_{div}^{minlen} = r^2 \int_0^{\ell/v} P_{div}(t)\, dt = \frac{2}{3}\frac{1}{\tau}\ell_d^3 \frac{d}{\lambda}\left(1 - 6\left(\frac{\ell_0}{\ell}\right)^2 + 4\left(\frac{\ell_0}{\ell}\right)^3\right) \tag{S34}$$

We choose the minimal loop size to be $\ell_0 = 3d$, since it is the minimal loop size required for an order of magnitude increase of the lifespan.

4.6 Immediate death of daughter loops slows down loop division.

Eq. (S14) also ignores the fact the newborn daughter loops have only one LEF and thus can die before becoming fully reinforced. This can happen even to large loops with the length $\ell > \ell_0$ and thus this effect is different from the one considered in the previous chapter.

The expression for the probability of successful division (S13) can be modified to take into account a chance of immediate death:

$$P_{div}^{imdeath}(\ell, t) = \frac{1}{\ell^2}\int_0^{\ell} dx_1 \int_0^{\ell} dx_2 \left(\mathbf{1}_{x_2 > x_1+vt} + \mathbf{1}_{x_2 < x_1-vt}\right)\left(1 - p_1^{death}(\ell_1)\right)\left(1 - p_1^{death}(\ell_2)\right)$$

The rate of loop division then can be calculated analytically if we truncate the expression for p^1_{death} (S28) at the second term:

$$p_1^{death}(\ell_d) \approx 1 - \frac{1}{1+\ell_d} = \frac{\ell_d}{\ell_d + 1}$$

$$\begin{aligned} R_{div}^{imdeath} &= r^2 \int_0^{\ell/v} P_{div}(\ell, t)\, dt = \\ &= \frac{2}{3}\frac{1}{\tau}\ell_d^3 \frac{d}{\lambda}\left(1 - 6\ell_d^{-1} - 6\ell_d^{-2} + 12\ell_d^{-3}\frac{(\ell_d+1)^2}{(\ell_d+2)}\ln(1+\ell_d)\right) \end{aligned} \tag{S35}$$

4.7 Loop size selection and immediate death affects daughter loops independently.

The most accurate expression for R_{div} should account both for the size selection and immediate death of daughter loops:

$$P_{div}^{full}(\ell, t) = \frac{1}{\ell^2} \int_0^\ell dx_1 \int_0^\ell dx_2 \left(\mathbf{1}_{x_2 > x_1 + vt} + \mathbf{1}_{x_2 < x_1 - vt} \right) \mathbf{1}_{\ell_1 > \ell_0} \mathbf{1}_{\ell_2 > \ell_0} \left(1 - p_1^{death}(\ell_1)\right) \left(1 - p_1^{death}(\ell_2)\right)$$

$$R_{div}^{full} = r^2 \int_0^{\ell/v} P_{div}^{full}(\ell, t)\, dt \tag{S36}$$

This expression does not have a short analytical form and has to be calculated numerically.

We compare the accuracy of the four different expressions for R_{div}(S14), (S34), (S35) and (S36) with the results of our simulations. For every tested combination of parameters (L, N, v, τ), we measure the rate of loop division R_{div} in the simulations and then estimate it with the equations (S14), (S34), (S35) and (S36) using the observed distribution of loop lengths (Fig. S12). We found that both corrections for loop size selection and immediate death of daughter loops significantly improve the accuracy of predicted R_{div}, with the combined expression (S36) having the highest accuracy.

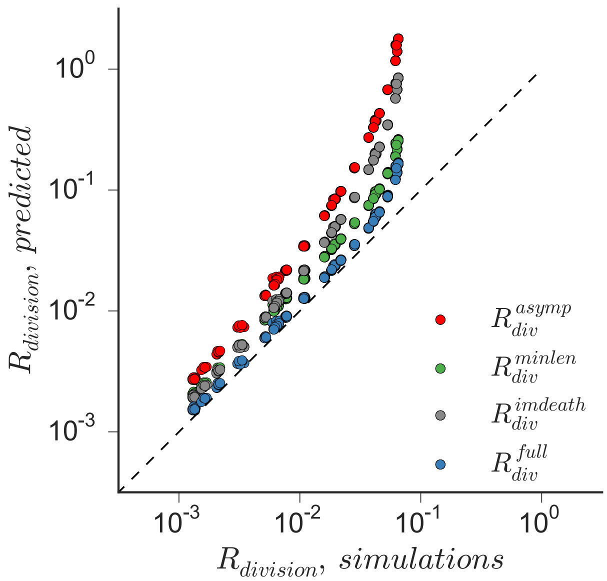

Figure S12: The rate of loop division in simulations can be predicted accurately using analytical expressions. The predictions with analytical expressions (S14), (S34), (S35) and (S36) are shown in red, green, gray and blue, correspondingly.

4.8 Fluctuations of the number of LEFs bound to the chromosome does not affect the properties of the steady state.

One of the simplifying assumptions of the LEF model is that, upon unbinding from chromatin, a LEF immediately rebinds to another randomly chosen site along the chromosome. Under this assumption, the number of LEFs bound to a chromosome stays constant throughout the simulation. To test whether this assumption affects the results of our simulations, we changed the model so that, upon unbinding, a LEF spends a random exponentially distributed period of time in the solution, and only then rebinds to the chromosome. In this new model, the average amount time spent by a LEF in the solution equals $\tau_{solution}$, the number of LEFs bound to chromosome at any point of time fluctuates around $N^* = \frac{\tau}{\tau + \tau_{solution}}$ and the adjusted value of the LEF separation is $d^* = {}^{L}/_{N^*}$. The simulations show that this advanced model of LEF rebinding does not change the properties of the steady state in either of the two regimes (appendix figure S13).

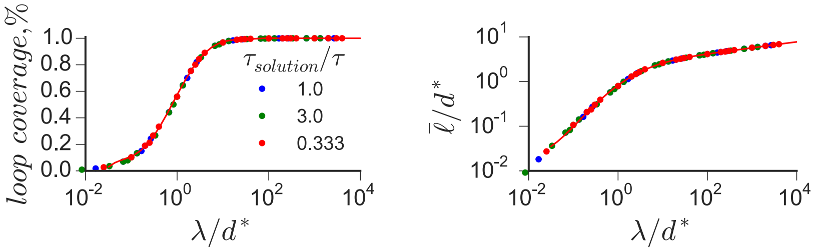

Figure S13: The loop coverage (left) and the normalized average loop length (right) in the simulations with delayed rebinding of LEFs. Using the adjusted value of LEF separation d^*, we show that these simulations perfectly agree with the simulations with immediate rebinding (the red line).

The simplified model and the model with a fluctuating number of bound LEFs are equivalent because the relative fluctuations of the number of LEFs on the chromosome and in the solution tend to zero as the system size tends to infinity, as it is was shown for grand canonical ensembles in statistical physics [8].

4.9 Closing gaps between the loops requires independent extrusion on the two sides of a LEF.

Another strong assumption made in the model is that a LEF extrudes two chromatin strands independently, such that blocking extrusion of one strand does not stop extrusion of the other. To test if this assumption is important for the observed effects of loop extrusion, we performed a set simulations using a modified model where blocking extrusion on one side of a LEF automatically blocked extrusion on the other side (Appendix figure S14).

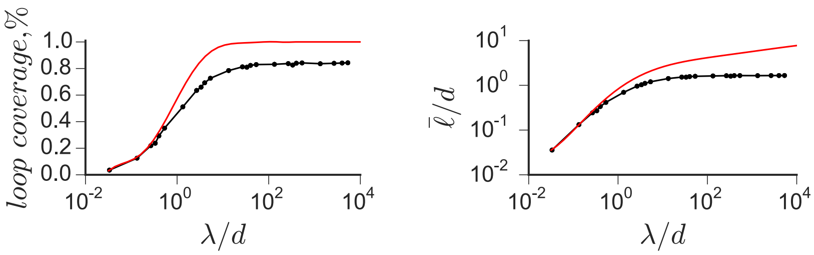

Figure S14: The loop coverage (left) and the average loop length (right) in simulations where blocking extrusion on one side of a LEF immediately blocks extrusion on the other. In the dense regime of this model, the gaps between loops do not close and the average loop size saturates at $\sim d$. The red line shows the results of the simulations in the standard model with independent extrusion of the two LEF heads.

The results of the modified simulations show that effective compaction requires independent extrusion of the two chromatin strands held by a LEF. In the modified model, the loop coverage saturates at ~85%, which corresponds to only ~6x lengthwise compaction (see Chapter 3.7). Coordinated blocking of extrusion also prevents the growth of the average loop size with λ/d and the average loop size saturates at $\sim d$. This result shows that, in order to efficiently compact a chromosome, LEFs must be able to move one of its contact points when the other is blocked.

5 Glossary and mathematical notation

- Loop extruding factor (LEF) - a molecular machine which bridges two adjacent sites on a chromosome and then moves the binding sites in the opposite directions along the chromosome, thus extruding a loop.
- Nested loop - a loop extruded inside some other loop (Fig.S15).
- Reinforced loop - a chromatin loop supported by several LEFs closely stacked on top of one another. Alternatively, a series of nested loops, formed by closely stacked LEFs.
- Branched loop - a loop containing two or more nested loops that are not nested into one another.
- Root loop - a loop that is not nested into any other loop; includes all nested loops, if it has any.
- L - the length of the chromosome.
- N - the number of LEFs in the system.
- τ - the average time that a LEF stays continuously bound to the chromosome.
- v - the average speed with which a LEF motor translocates chromatin fiber.
- λ - LEF processivity, the average length of a chromatin loop that a single unobstructed LEF can extrude over its residence time.
- d - LEF separation, the average spacing between LEFs if they were randomly dispersed along the chromosome.
- a - the thickness of the fiber.
- n - the number of LEFs supporting a reinforced loop.
- ℓ - the length of a loop.
- ℓ_d - the length of a loop normalized by the LEF separation.
- $\overline{\ell}$ - the average length of a loop in the steady state.
- $\overline{\ell}_d$ - the average length of a loop in the steady state normalized by the LEF separation.
- $\overline{n}$ - the average number of LEFs supporting a reinforced loop in the steady state.
- r - the rate of LEF binding per loop.
- μ - the rate of LEF unbinding.

- f - the fraction of the chromosome contained in the gaps between the loops.
- c_{len} - the coefficient of the lengthwise compaction.
- R_{death} - the rate of death of reinforced loops.
- R_{div} - the rate of division of reinforced loops.
- t_a^{opt} - the optimal rate of stochastic LEF activation that provides the fastest convergence to the steady state.

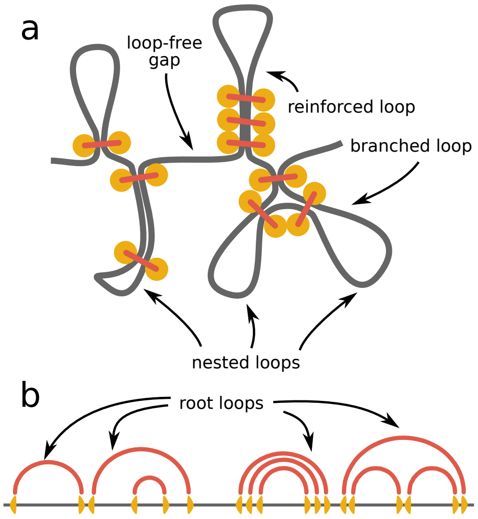

Figure S15: (a) The illustration of the possible loop structures formed by loop-extruding factors (LEFs). (b) The corresponding diagram of the intramolecular links formed by LEFs.

References

[1] Elnaz Alipour and John F Marko. Self-organization of domain structures by DNA-loop-extruding enzymes. *Nucleic acids research*, 40(22):11202–12, December 2012.

[2] Kiichi Fukui and Susumu Uchiyama. Chromosome protein framework from proteome analysis of isolated human metaphase chromosomes. *Chemical record (New York, N.Y.)*, 7(4):230–7, January 2007.

[3] Ai Takemoto, Keiji Kimura, Shigeyuki Yokoyama, and Fumio Hanaoka. Cell cycle-dependent phosphorylation, nuclear localization, and activation of human condensin. *The Journal of biological chemistry*, 279(6):4551–9, February 2004.

[4] Robert G. Gallager. *Stochastic Processes: Theory for Applications*. Cambridge University Press, 2013.

[5] Mark A. Pinsky and Samuel Karlin. *An Introduction to Stochastic Modeling*. Academic Press, 2011.

[6] Natalia Naumova, Maxim Imakaev, Geoffrey Fudenberg, Ye Zhan, Bryan R Lajoie, Leonid a Mirny, and Job Dekker. Organization of the mitotic chromosome. *Science (New York, N.Y.)*, 342(6161):948–53, November 2013.

[7] Daniel Gerlich, Toru Hirota, Birgit Koch, Jan-Michael Peters, and Jan Ellenberg. Condensin I stabilizes chromosomes mechanically through a dynamic interaction in live cells. *Current biology : CB*, 16(4):333–44, February 2006.

[8] Mehran Kardar. *Statistical Physics of Particles*. Cambridge University Press, June 2007.
