# Supplementary figures and images for: Chromosome Compaction by Active Loop Extrusion
Source: Biophys J. 2016 May 24;110(10):2162–8. doi: 10.1016/j.bpj.2016.02.041 (PMC4880799; doi:10.1016/j.bpj.2016.02.041)

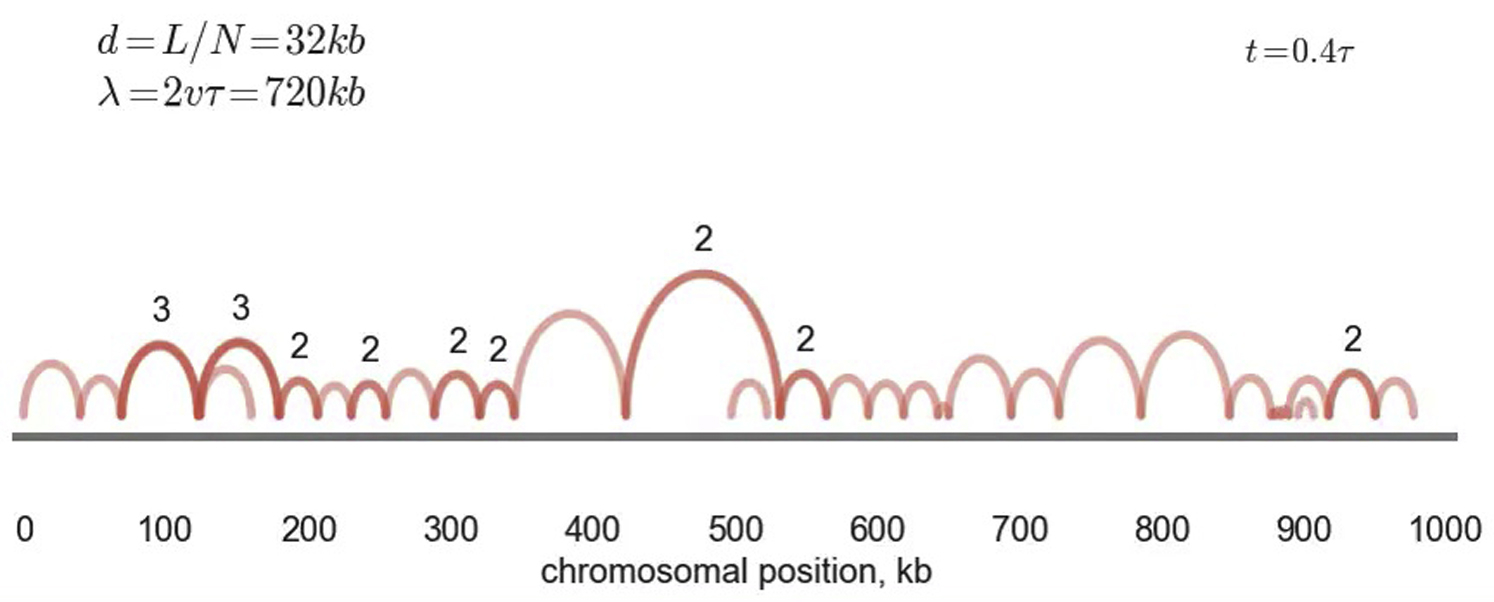

Supplement: Movie S1. Representative Simulation of Chromosome Compaction by Loop Extruding Factors [file mmc2.jpg]
